# More Questions than Answers: Continued Critical Reanalysis of Fredrickson et al.’s Studies of Genomics and Well-Being

**DOI:** 10.1371/journal.pone.0156415

**Published:** 2016-06-07

**Authors:** Nicholas J. L. Brown, Douglas A. MacDonald, Manoj P. Samanta, Harris L. Friedman, James C. Coyne

**Affiliations:** 1 University Medical Center, Groningen, The Netherlands; 2 University of Detroit Mercy, Detroit, Michigan, United States of America; 3 Systemix Institute, Redmond, Washington, United States of America; 4 Goddard College, Plainfield, Vermont, United States of America; 5 University of Florida, Gainesville, Florida, United States of America; University of Illinois-Chicago, UNITED STATES

## Abstract

We critically re-examine Fredrickson et al.’s renewed claims concerning the differential relationship between hedonic and eudaimonic forms of well-being and gene expression, namely that people who experience a preponderance of eudaimonic well-being have gene expression profiles that are associated with more favorable health outcomes. By means of an extensive reanalysis of their data, we identify several discrepancies between what these authors claimed and what their data support; we further show that their different analysis models produce mutually contradictory results. We then show how Fredrickson et al.’s most recent article on this topic not only fails to adequately address our previously published concerns about their earlier related work, but also introduces significant further problems, including inconsistency in their hypotheses. Additionally, we demonstrate that regardless of which statistical model is used to analyze their data, Fredrickson et al.’s method can be highly sensitive to the inclusion (or exclusion) of data from a single subject. We reiterate our previous conclusions, namely that there is no evidence that Fredrickson et al. have established a reliable empirical distinction between their two delineated forms of well-being, nor that eudaimonic well-being provides any overall health benefits over hedonic well-being.

## Introduction

Fredrickson et al. [1] claimed to have established that individuals who experience a preponderance of hedonic over eudaimonic well-being will have different “Conserved Transcriptional Response to Adversity” (CTRA) gene expression profiles compared to those who have the inverse preponderance. Fredrickson et al. reported that these CTRA profiles differed such that higher hedonic well-being was associated with increased expression of pro-inflammatory genes, typically seen in immune responses to bacterial infections, whereas higher eudaimonic well-being was associated with increased expression of genes involved in type I interferon antiviral responses and IgG1 antibody synthesis (henceforth: “antiviral” genes), typically seen in responses to viral threats. In a previous article [2], we demonstrated that Fredrickson et al.’s [1] study was flawed in several ways, including misidentification of hedonic and eudaimonic well-being factors, questionable data analytic methods based on the unjustified aggregation of non-significant regression coefficients, and an elementary but crucial error in the coding of their dataset.

Recently, Fredrickson et al. [3] published a follow-up article in which they claimed to have reproduced and extended the results from their initial study [1], and to have refuted most of the criticisms made by us in our previous article [2]. However, we show here that this follow-up article is also flawed in several ways. First, it suffers from the same problems of scale validity as the first article. Second, Fredrickson et al.’s new data analysis model, when applied to their original data, gives results that are inconsistent with those from their previous model, contradicting their earlier conclusions [1]; this new model also gives results that differ between their new sample and their original sample, contradicting the claim that their more recent study [3] is a successful replication and extension of their earlier study [1]. Third, it seems that there has been a substantial (but unacknowledged) shift in the hypotheses across the two articles [1, 3]. Fourth, we show that Fredrickson et al. used two versions of the same dataset at different points in their new article [3]. Fifth, a recently-discovered issue demonstrates that both of Fredrickson et al.’s [1, 3] models are very sensitive to the presence of outliers in their dataset. We conclude that these problems make Fredrickson et al.’s results uninterpretable.

## Factor Structure of the MHC-SF

We previously noted [2] a number of serious flaws with the way in which Fredrickson et al. [1] measured the crucial distinction between hedonic and eudaimonic well-being in their first article, including the very high intercorrelation between the two measures; the demonstration that the factor structure of the Mental Health Continuum-Short Form (MHC-SF) scale, both in previous independent studies and Fredrickson et al.’s own sample, did not accord with their interpretation of it; and the fact that the references cited by Fredrickson et al. [1, 3] as supporting their claims of a two-factor structure did not in reality do so.

We ran essentially the same analyses as before [2] to test the factor structure of the MHC-SF using the confirmation sample (n = 122) from Fredrickson et al. [3] to determine if problems similar to those that we had found and reported previously [2, 4] were still present. Our results, presented in more detail in the Supporting Information, (S1 File), point to continued problems with the measurement of well-being using the MHC-SF; in particular, there is a lack of strong support for two factors that could be labeled “hedonic” and “eudaimonic” well-being. Thus, any assertions about the differential effect of hedonic and eudaimonic well-being on genomic variables based on this measure do not appear to be justified. Indeed, other research [5] suggests that these two forms of well-being are, empirically, essentially indistinguishable from each other.

## The Mixed Effect Linear Model

Fredrickson et al. [3] were strongly critical of our reanalysis [2] of the regression method used in their initial study [1], which we named “RR53.” In that reanalysis, we demonstrated—by evaluating all possible “factor” pairs (that is, all possible ways of splitting the 14 MHC-SF items into two distinct and non-empty sets)—that the RR53 method would generate statistically significant results, apparently indicating a meaningful differential association of well-being with gene expression, for almost any “factors,” and that this effect was repeated—with only a slightly lower rate of spurious matches—when the MHC-SF item data were replaced by random numbers. Subsequently, other authors ([6, 7]) have suggested that the principal flaw in the RR53 regression method lies in its application of a *t* test to non-independent data, namely the collected coefficients from multiple separate regressions of the expression of individual genes on measures of well-being. However, Fredrickson et al. [3] described our systematic, exhaustive examination of the functioning of their method as “capitaliz[ing] on chance” (p. 11). Despite their rejection of our criticism of their RR53 method, Fredrickson et al. replaced it with a mixed effect linear model to perform the majority of their analyses in their later article [3]. They applied this model to several datasets. First, they analyzed new data (which they named the “confirmation sample”) collected from a new sample of 122 individuals following the methods and protocols described in their original study [1]. Second, they applied their new model to an aggregate (“pooled”) dataset, which augmented the confirmation sample with the dataset from their original study [1] (the “discovery sample”). Third, they analyzed an additional dataset (the “generalization sample”), apparently taken from a different study that used a different psychometric instrument (the Ryff Scales of Psychological Well-Being [8]).

The implementation of Fredrickson et al.’s [3] new model in SAS, as applied to the confirmation and pooled samples, was extensively documented in Fredrickson et al.’s supplemental materials, so we had little difficulty in reproducing it in R, using the **gls()** command from the **nlme** package. Our reproduction produced results that were identical to those reported by Fredrickson et al. [3] in their Tables 2 and 3, within the limits of rounding error (see Table C in S1 File). We therefore felt confident that our model was a faithful replication of the one built in SAS by Fredrickson et al. (As we were unable to reproduce the model used to analyze the generalization sample, we do not examine that sample further here.)

Fredrickson et al. [3] did not report the results of the application of their new model to the discovery sample (i.e., the dataset from their first study [1]) on its own. Yet, if their aim was to show that their new study had confirmed the results of the first—for which they claimed support with their Fig 1C, showing that their original model produced “similar” results with their new sample—it would seem logical to demonstrate that their new model also produced comparable results when applied to the original sample. We therefore applied our replication of Fredrickson et al.’s [3] model to the discovery study data [1]. Here, we found a very different pattern of effects of hedonic and eudaimonic well-being on gene expression, compared to the results obtained by Fredrickson et al. [3] in their analysis of the confirmation sample. In the latter analysis, Fredrickson et al. noted that the association between hedonic well-being and gene expression was small and non-significant (*b* = .085, *p* = .4829), whereas the association for eudaimonic well-being and gene expression was substantial and significant (*b* = −.509, *p* < .0001). However, our analyses of the discovery sample show precisely the opposite effects. That is, in the discovery sample, hedonic well-being was substantially and significantly associated with CTRA gene expression (*b*s from .490 to .568, *p*s from .0031 to .0009), whereas the association between eudaimonic well-being and gene expression was not significant (*b*s from .134 to .195, *p*s from .2588 to .4522). Thus, the application of the mixed effect linear model to the two individual samples (discovery and confirmation) gives results that are diametrically opposed to each other; yet, only one of these sets of results (for the confirmation study) was reported by Fredrickson et al. [3], who instead chose to report the results of combining the discovery and confirmation samples into one aggregate (“pooled”) sample. Our analyses of the discovery sample using the mixed effect linear model are presented in detail, and contrasted with Fredrickson et al.’s analysis of the confirmation sample, in the Supporting Information (Table D in S1 File).

**Fig 1:**
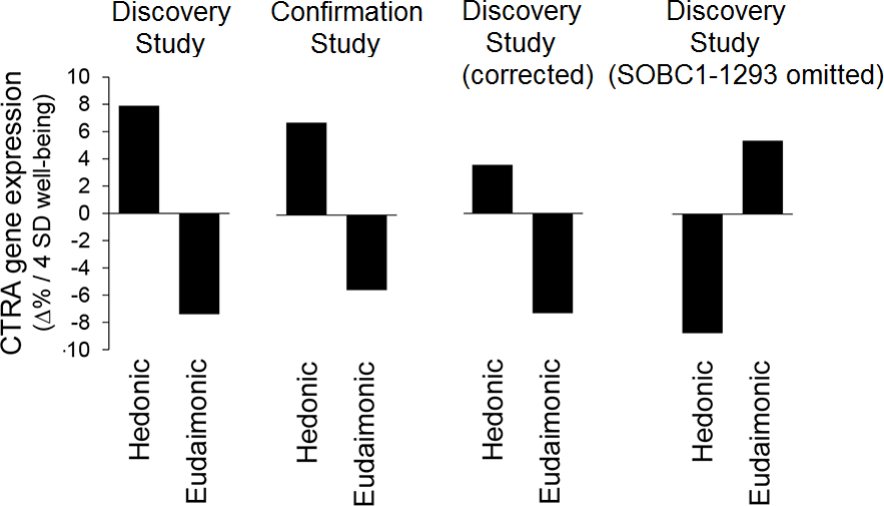
Extensions of Fredrickson et al.’s [2] Figure 1C. In this figure, we have augmented Fredrickson et al.’s [2] Fig 1C—from which the first two pairs of bars in our figure are directly taken—with two new pairs of bars. The third pair from the left, labeled “Discovery Study (corrected),” indicates the fold difference values that are produced by the application of Fredrickson et al.’s [1] repeated-regression method to the discovery sample, after correction of the variable “White” for participant SOBC1-1299 from “4” to “0”. The rightmost pair, labeled “Discovery Study (SOBC1-1293 omitted),” indicates the fold difference values produced by the omission of participant SOBC1-1293 (cf. discussion in the text of the present article).

## Apparent Change of Hypotheses

In their first article on this topic, Fredrickson et al. [1] consistently reported that hedonic well-being was associated with up-regulation of pro-inflammatory genes and down-regulation of antiviral genes, with the opposite associations applying to eudaimonic well-being. Almost every mention of either form of well-being (hedonic or eudaimonic) in that article was immediately counterbalanced by a mention of the other form, accompanied by a demonstration of an inverse relationship with CTRA gene expression. This symmetry was a recurring theme throughout the whole article.

In their Fig 1C at the start of their more recent article [3], Fredrickson et al. appeared to show that the application of their original regression model [1] to their new dataset (the “confirmation” sample) reproduced the results of their initial study—with up-regulation of pro-inflammatory CTRA genes being associated with hedonic well-being and up-regulation of antiviral genes being associated with eudaimonic well-being. However, the rest of this newer article appeared to espouse a revised version of their theory, in which there is a purported relationship between (only) eudaimonic well-being and CTRA gene expression, with no role for hedonic well-being. No reasons were provided for this change in theoretical direction; indeed, Fredrickson et al. appeared to suggest that no change had even taken place:

The present findings thus *converge with previous results* [emphasis added] in identifying eudaimonic well-being as the primary source of associations between overall well-being and CTRA gene expression and provide no support for any independent favorable contribution from hedonic well-being. The consistency and robustness of these findings across 3 independent study samples also refutes claims that the initial discovery study findings were somehow spurious or unreplicable. ([3], p. 9)

However, at no point in their earlier article [1] did Fredrickson et al. assert that eudaimonic well-being was the “primary source of associations” between well-being and gene expression. Indeed, as noted above, that article treated hedonic and eudaimonic well-being as symmetrical and of equivalent importance, with the abstract stating, for example, that “hedonic and eudaimonic well-being showed…highly divergent transcriptome profiles” and that these two forms of well-being “engage distinct gene regulatory programs” ([1], p. 13684).

A careful reading of the paragraph quoted above suggests that what the reader is invited to understand here is that only eudaimonic well-being produces “favorable” changes in CTRA gene expression, presumably through the activation of (only) antiviral genes. This implies that the symmetric (and, presumably, “unfavorable”) association of the expression of pro-inflammatory genes with hedonic well-being, identified by Fredrickson et al. in their initial analyses of their discovery sample [1], should now be ignored. Such a change of interpretation would also require the reader to ignore our demonstration (above) that the application of Fredrickson et al.’s new analysis method (their mixed effect linear model [3]) to the same discovery sample shows that only hedonic (and not eudaimonic) well-being had significant associations with gene expression in that sample. Thus, an examination of the complete available evidence suggests that Fredrickson et al.’s preferred conclusion is by no means the only one supported by it.

## Multiple Versions of the Discovery Sample Dataset

Fredrickson et al. [3] used their Fig 1C to present the “[p]oint estimates of average association coefficients relating range-spanning variations in hedonic and eudaimonic well-being scores [−2 SD, +2 SD] to unstandardized (log_2_ metric) gene expression values” (p. 5) in their discovery and confirmation studies. In other words, this figure represents the results of applying their original regression method from [1] to both their initial (n = 76) “discovery” dataset [1] and their new (n = 122) “confirmation” dataset [3], and, at first glance, appears to show that these results are similar. However, a closer analysis reveals two substantial problems here.

First, as we have previously demonstrated [2, 4], Fredrickson et al.’s original regression method finds large numbers of spurious associations with opposing signs for hedonic and eudaimonic well-being where no real effect exists, even when random numbers are used in place of their psychometric data. Thus, it is no surprise that the application of this method to their new data appears to produce similar results.

Second, Fredrickson et al.’s original analysis [1] was greatly distorted by one participant (with identification code SOBC1-1299) having an incorrectly coded value of “4” for the variable “White” (a binary indicator of the participant’s race). This error effectively transformed this categorical variable into a continuous variable, with dramatic consequences for the regressions in which it was used. When we re-ran our reproduction of Fredrickson et al.’s regression method [1] after correcting the value of “White” for this participant from “4” to “0”, the fold-difference value for hedonic well-being changed from +8.0% to +3.6%. This elementary dataset coding error—which we reported in some detail in our first critique [2, 4]—ought to have been corrected before Fig 1C [3] was generated, but the *y*-axis of that figure shows that it was not (see also our Fig 1). However, it appears that Fredrickson et al. *did* correct this error when constructing their “pooled” dataset used for the principal analyses (with their new mixed effect linear model) shown in their Table 3 [3]; our reproduction of Fredrickson et al.’s new model [3] gives results that are closer to the numbers in their Table 3 when the dataset coding error is indeed corrected. This means that Fredrickson et al. [3] in effect *used two different versions* of the GSE45330 dataset in their article [3]; the first (with SOBC1-1299 “White” having the uncorrected value of “4”) was used to demonstrate that their original regression model produced similar results with both the discovery and confirmation samples (as shown in their Fig 1C), while the second (with SOBC1-1299 “White” corrected to “0”) was used as input to their Table 3, as part of the pooled study.

We note that Fredrickson et al. have recently issued a correction [9] to their Fig 1C, although in so doing they did not amend the text from their article [3] in which they described the two halves of this figure as showing “similar magnitudes of association between unstandardized (log2) CTRA gene expression and well-being scores” (p. 6). Given that the height of the bar for the association between hedonic well-being and gene expression in the discovery sample has been reduced by 60% as a result of this correction and is now only half the height of the corresponding bar for the confirmation sample, we question whether this claim of similarity is still valid.

## Sensitivity of Analysis Methods to Individual Data Points

A preliminary analysis of any dataset should always include an evaluation of the extent to which outliers may be driving the results [10], even if the subsequent question of whether to exclude a given data point or participant is often a subjective one. The number of independent variables in Fredrickson et al.’s models [1, 3] makes it difficult to determine visually (e.g., using a scatterplot) whether any individual participant’s data might be having an undue influence on the overall outcome. We therefore ran these models multiple times, omitting one participant each time, to see whether this would make a disproportionate change to the results. We discovered that the omission of one participant (reference number SOBC1-1293, a male) caused several of the purported associations between gene expression and both hedonic and eudaimonic well-being to *change sign*, and the remainder to undergo a substantial change in magnitude, whether Fredrickson et al.’s original RR53 regression method or their new mixed effect linear model was used. Specifically, in the case of the RR53 method, the fold difference value for hedonic well-being changes from +3.6% to −8.8% when this participant is excluded, while the value for eudaimonic well-being changes from −7.3% to +5.3%. In the case of the mixed effect linear model, the *b*-coefficients change from 0.536 to −0.120 (hedonic) and from 0.135 to 0.065 (eudaimonic). (See Table E in S1 File for full details of this analysis.)

This improbable-sounding observation is a major problem for several reasons. First, and most obviously, the presence of any given individual in a study where participants were recruited from the community via flyers and e-mail [1] is, essentially, a random event. Had participant SOBC1-1293 not enrolled in the initial study, Fredrickson et al. [1] would presumably have reached diametrically opposite conclusions about the relative effects of hedonic and eudaimonic well-being on CTRA gene expression. Second, closer inspection shows that this participant’s data were in many respects unusual, thus making him a good candidate for exclusion as an outlier; for example, he gave answers at the extreme ends of the scale (“0” or “5”) for every item in the MHC-SF, and a substantial number of his gene expression values were near the extremes for the sample. Third, the large differences in the results of their analyses depending on whether this one participant is included or excluded—illustrated in the right-hand panel of Fig 1—demonstrates that both of Fredrickson et al.’s models (the RR53 regression procedure [1] and the mixed effect linear model [3]) are highly sensitive to minor variations in the input data. It has been suggested that this sensitivity may be due to overfitting, caused by the combination of a large number of predictors and a small sample size [11].

## Conclusion

We believe that Fredrickson et al. have not provided any substantial evidence to support their hypotheses regarding the relationship between well-being and gene expression, which also appear to have changed across their two articles [1, 3]. In their initial study, they reported that both hedonic and eudaimonic well-being were differentially and symmetrically associated with opposite forms of CTRA gene regulation; we showed that their theoretical approach, factor analysis, and regression methods suffered from a variety of problems, and we have now demonstrated that the principal result in that initial article were heavily dependent on the data from a single participant. In their latest article [3], Fredrickson et al. presented new evidence for what appear to be a different set of hypotheses (i.e., that only eudaimonic well-being is associated with gene expression), while not addressing many of the issues that we raised in our previous critique [2] which also apply to this latest article. They changed their statistical model to one which, when applied to their original dataset, produced results that were in contradiction with both their conclusions from applying their original model [1] and the results obtained when applying this new model to their new sample [3]. As shown above, they also utilized two versions of the same dataset within the same PLoS ONE article. All of this methodological confusion makes it impossible to place any meaningful interpretation on their results. To put it as simply as possible: Fredrickson et al.’s two studies [1, 3] provide no clear and uncontestable evidence that higher levels of eudaimonic, as compared to hedonic, well-being is associated with healthier patterns of gene expression.

## Supporting Information

S1 File Supporting Information. (PDF)
