# Supplementary material for: More Questions than Answers: Continued Critical Reanalysis of Fredrickson et al.’s Studies of Genomics and Well-Being
Source: PLoS One. 2016 Jun 7;11(6):e0156415. doi: 10.1371/journal.pone.0156415 (PMC4896417; doi:10.1371/journal.pone.0156415)
Supplement: S1 File — (PDF) [file pone.0156415.s001.pdf]

Supporting Information

Factor analysis of the MHC-SF in the confirmation sample

We completed principal axis and maximum likelihood exploratory factor analyses (EFA) using both statistical (eigenvalue > 1) and rational (extract two factors) extraction rules, with both orthogonal and oblique (promax) factor rotations. Thereafter, we tested four different measurement models using confirmatory factor analysis (CFA) with maximum likelihood estimation. The models were one-factor (i.e., all 14 MHC-SF items loading on a unitary general well-being factor), correlated two-factor (using hedonic and eudaimonic factors with item assignment consistent with Fredrickson et al.'s first study [1]), and two different correlated three-factor models, namely the model reported by Keyes [2] which has emotional, psychological, and social well-being factors, and the three-factor model we had previously identified [3, 4] which has hedonic and eudaimonic factors along with an additional factor that we labeled "evaluative perception of the social environment." Although some differences were found in terms of exact patterns of elevated loadings in the EFAs, the results overall point to continued problems with conceptualization and measurement of well-being using the MHC-SF. Specifically, we found that (a) EFA solutions only accounted for little more than half of the score variance (about 56%), (b) obliquely rotated EFA factors were highly intercorrelated (e.g., $r = .73$) and estimated factor correlations in two- and three-factor CFA models were very elevated and significant (e.g., correlations ranged from .82 in the two-factor model to as high as .93 between the psychological and social factors in Keyes' [2] three-factor model), and (c) regardless of the number of factors specified in CFA models, there was evidence of inadequate model fit. In short, the MHC-SF does not appear to be measuring what Fredrickson et al. claim it to be measuring. Any assertions about the differential effect of hedonic and eudaimonic well-being on genomic variables based on this

measure cannot be justified. The results of all our exploratory and confirmatory analyses can be seen in our Table A and Table **B**.

Table A. Descriptive Statistics for Mental Health Continuum Short Form (MHC-SF) Items and Exploratory Factor Analytic Results

			Statistical Extraction Criterion (R >1) Varimax Rotation				Rational Extraction Criterion (2 factors) Varimax Rotation				Statistical Extraction Criterion Principal Axis Factor Oblique Rotation				Rational Extraction Criterion Maximum Likelihood Factor Oblique Rotation			
			PAF		MLF		PAF		MLF		Pattern		Structure		Pattern		Structure	
MHCSF Item	Mean	SD	1	2	1	2	1	2	1	2	1	2	1	2	1	2	1	2
1	3.68	1.17	.27	**.76**	.29	**.78**	.27	**.76**	.29	**.78**	−.08	**.86**	**.55**	**.80**	−.06	**.87**	**.58**	**.83**
2	3.92	1.18	.21	**.87**	.24	**.88**	.21	**.87**	.24	**.88**	−.22	**1.05**	**.55**	**.89**	−.18	**1.04**	**.58**	**.91**
3	3.30	1.30	**.44**	**.68**	**.48**	**.64**	**.44**	**.68**	**.48**	**.64**	.19	**.66**	**.67**	**.80**	.28	**.57**	**.69**	**.77**
4	3.32	1.52	**.61**	**.56**	**.64**	**.52**	**.61**	**.56**	**.64**	**.52**	**.49**	.39	**.78**	**.75**	**.56**	.31	**.79**	**.72**
5	3.20	1.69	**.47**	**.50**	**.51**	**.45**	**.47**	**.50**	**.51**	**.45**	.34	**.40**	**.63**	**.65**	**.43**	.30	**.65**	**.62**
6	1.62	1.42	**.50**	.31	**.47**	.32	**.50**	.31	**.47**	.32	**.50**	.12	**.59**	**.49**	**.45**	.15	**.56**	**.48**
7	3.31	1.28	**.76**	.12	**.73**	.12	**.76**	.12	**.73**	.12	**.95**	−.29	**.74**	**.41**	**.91**	−.27	**.71**	.39
8	2.06	1.53	**.59**	.22	**.56**	.23	**.59**	.22	**.56**	.23	**.67**	−.06	**.63**	**.44**	**.62**	−.02	**.60**	**.42**
9	3.43	1.33	**.71**	**.40**	**.75**	.35	**.71**	**.40**	**.75**	.35	**.72**	.12	**.81**	**.65**	**.81**	.02	**.83**	**.61**
10	3.07	1.35	**.63**	**.47**	**.66**	**.43**	**.63**	**.47**	**.66**	**.43**	**.58**	.25	**.76**	**.68**	**.64**	.18	**.78**	**.65**
11	3.72	1.29	**.51**	**.58**	**.57**	**.53**	**.51**	**.58**	**.57**	**.53**	.35	**.48**	**.71**	**.74**	**.46**	.37	**.73**	**.71**
12	3.22	1.43	**.52**	**.41**	**.52**	**.41**	**.52**	**.41**	**.52**	**.41**	**.46**	.24	**.64**	**.58**	**.46**	.25	**.64**	**.58**
13	3.77	1.24	**.63**	**.44**	**.68**	**.40**	**.63**	**.44**	**.68**	**.40**	**.60**	.21	**.75**	**.65**	**.69**	.13	**.78**	**.63**
14	3.20	1.63	**.61**	**.42**	**.64**	.37	**.61**	**.42**	**.64**	.37	**.58**	.20	**.72**	**.62**	**.65**	.12	**.74**	**.59**
% Variance Explained			52.0	5.4	51.5	5.8	52.0	5.4	51.5	5.8	52.0	5.4			51.5	5.8		

Note. N = 122. For the MHC-SF, item scores can range from 0 to 5. Initial data screening revealed item response data complete for all participants save item 12 for Case 2073. Missing response was imputed with mean score calculated from remaining 13 items for this case. Examination of kurtosis and skewness did not indicate severe problems with non-normality. PAF= Principal Axis Factor Analysis. MLF= Maximum Likelihood Factor Analysis. For obliquely rotated factors, promax rotation was used. For both obliquely rotated PAF and MLF solutions, factor correlation= .73. For all three maximum likelihood factors χ^2 = 124.11, df= 64, $p<.001$. All loadings .40 or higher are in bold font.

Table B. Confirmatory Factor Analysis Results for Four Models Tested

	One Factor	Two Factor (as per Fredrickson et al 1)		Three Factor (as per Keyes 5)			Three Factor (as per Brown et al 3)		
MHCSF item		Hed	Eud	Emo	Soc	Psy	Hed	Eud	EPSE
1	.70	.85	---	.85	---	---	.84	---	---
2	.73	.85	---	.85	---	---	.86	---	---
3	.78	.81	---	.81	---	---	.81	---	---
4	.83	---	.83	---	.86	---	---	.83	---
5	.69	---	.70	---	.72	---	---	.70	---
6	.57	---	.56	---	.60	---	---	---	.69
7	.62	---	.65	---	.64	---	---	---	.74
8	.57	---	.57	---	.59	---	---	---	.71
9	.79	---	.81	---	---	.82	---	.81	---
10	.78	---	.78	---	---	.79	---	.78	---
11	.78	---	.78	---	---	.78	---	.78	---
12	.66	---	.67	---	---	.67	---	.67	---
13	.77	---	.78	---	---	.79	---	.78	---
14	.73	---	.74	---	---	.74	---	.74	---
Fit Indices									
χ^2	211.18 (p<.001)	166.49 (p<.001)		158.15 (p<.001)			145.43 (p<.001)		
df	77	76		74			74		
GFI	.80	.85		.85			.86		
CFI	.87	.91		.92			.93		
RMSEA	.120 (p<.001)	.099 (p<.001)		.097 (p<.001)			.089 (p<.01)		
Comparison of models									
1-factor	---	$\Delta \chi^2$ = 44.69 Δdf = 1, p<.001		$\Delta \chi^2$ = 53.03, Δdf = 3, p<.001			$\Delta \chi^2$ = 65.75, Δdf = 3, p<.001		
2-factor	---	---		$\Delta \chi^2$ = 8.34, Δdf = 2, p<.05			$\Delta \chi^2$ = 21.06, Δdf = 2, p<.001		

Note. N= 122. "Hed"= Hedonic, "Eud"= Eudaimonic, "Emo"= Emotional, "Soc"= Social, "Psy"= Psychological, "EPSE"= Evaluative Perception of the Social Environment. For all models, maximum likelihood estimation was used. Standardized regression weights are reported in the table. Parameters for all models (e.g., regression weights, item error variances, and factor correlations) were found to be significant at $p < .05$ or lower. For 2-factor model, factor correlation= .82. For Keyes et al. 3-factor model, factor correlations as follows: Emo-Soc= .80; Emo-Psy= .81; Soc-Psy= .93. For Brown et al. 3-factor model, factor correlations were as follows: Hed–Eud= .83; Hed–EPSE= .62; Eud–EPSE= .81. For all four models, bootstrap analyses using 1000 samples were completed and suggested that parameter estimates are reasonably robust. For difference-in-chi-square comparisons across models, no comparison was made between the two 3-factor models due to the fact that there is no difference in degrees of freedom (df) across the two models. These comparisons indicate that the 1-factor model demonstrates worse fit than 2- and 3-factor models and that the 2-factor model demonstrates worse fit than both 3-factor models.

Table C Calibration of our reproduction of Fredrickson et al.'s mixed effect linear model[1].

Study	Well-being model	Well-being dimension	Association *b*	SE	Test statistic	p	VIF
Confirmation (n = 122)							
	2-dimensional						
		Hedonic well-being	0.086 / *0.085*	0.122 / *0.121*	*t*(104) = 0.70 / *0.70*	.4820 / *.4829*	2.46 / *2.46*
		Eudaimonic well-being	−0.511 / *−0.509*	0.125 / *0.125*	*t*(104) = −4.08 / *−4.08*	<.0001 / *<.0001*	2.59 / *2.59*
	3-dimensional						
		Hedonic well-being	0.098 / *0.098*	0.122 / *0.122*	*t*(103) = 0.80 / *0.80*	.4232 / *.4243*	2.49 / *2.49*
		Psychological well-being	−0.385 / *−0.384*	0.154 / *0.153*	*t*(103) = −2.50 / *−2.50*	.0125 / *.0139*	3.95 / *3.95*
		Social well-being	−0.163 / *−0.163*	0.137 / *0.137*	*t*(103) = −1.19 / *−1.19*	.2350 / *.2370*	3.14 / *3.14*
	1-dimensional						
		Total well-being	−0.441 / *−0.439*	0.086 / *0.086*	*t*(105) = −5.11 / *−5.11*	<.0001 / *<.0001*	1.17 / *1.17*
	Categorical						
		Flourishing	−0.615 / *−0.615*	0.176 / *0.176*	*t*(105) = −3.49 / *−3.49*	.0005 / *.0007*	1.17 / *1.17*
		Flourishing (corrected)[2]	−0.693 / ——	0.178 / ——	*t*(105) = −3.89 / ——	.0001 / ——	1.17 / ——
Pooled (n = 198)							
	2-dimensional						
		Hedonic well-being	0.074 / *0.074*	0.042 / *0.042*	*t*(179) = 1.77 / *1.77*	.0767 / *.0781*	2.44 / *2.44*
		Eudaimonic well-being	−0.116 / *−0.115*	0.043 / *0.043*	*t*(179) = −2.71 / *−2.71*	.0068 / *.0074*	2.55 / *2.54*
	3-dimensional						
		Hedonic well-being	0.059 / *0.059*	0.042 / *0.042*	*t*(178) = 1.39 / *1.39*	.1650 / *.1663*	2.53 / *2.53*
		Psychological well-being	0.015 / *0.015*	0.052 / *0.052*	*t*(178) = 0.29 / *0.29*	.7700 / *.7702*	3.87 / *3.82*
		Social well-being	−0.126 / *0.126*	0.045 / *0.045*	*t*(178) = −2.81 / *−2.81*	.0050 / *.0055*	2.84 / *2.83*
	1-dimensional						
		Total well-being	0.050 / *0.050*	0.028 / *0.028*	*t*(180) = 1.77 / *1.78*	.0761 / *.0775*	1.09 / *1.07*
	Categorical						
		Flourishing	−0.051 / *−0.051*	0.056 / *0.056*	*t*(180) = −0.91 / *−0.91*	.3637 / *.3628*	1.08 / *1.06*
		Flourishing (corrected)	0.016 / ——	0.057 / ——	*t*(180) = −0.28 / ——	.7824 / ——	1.09 / ——

[1] The table entries in roman type show the results from our reproduction model; those in italics are taken from Fredrickson et al.'s [5] Tables 2 and 3.

[2] It appears that Fredrickson et al. [5] incorrectly calculated the value of categorical flourishing for a number of participants. According to Keyes [2] and as noted by Fredrickson et al. [5] (Table 1, footnote 2), to be considered as flourishing, a participant needs to endorse experiencing at least one of three "hedonic" items either "every day" or "almost every day"—counted as a score of 5 or 4, respectively, on one of the MHC-SF items SF1–SF3—and also to endorse experiencing at least six of eleven "positive functioning" items (SF4–SF14) with the same frequency. However, for eight participants in the discovery study and seven participants in the dataset for the confirmation study, the variable "FlourishGroup" was set to "1", although the number of positive functioning items endorsed with a score of 4 or 5 was only five, not six. These participants were thus categorized as flourishing when they did not meet the criteria defined in [2] (see also note 2 on Table 2 in [5]). In our reproduction of Fredrickson et al.'s Tables 2 and 3, and our subsequent analysis of the discovery sample, we calculated values for both the incorrect (five or more positive functioning items endorsed with a score of 4 or 5) and the correct (six or more positive functioning items endorsed with a score of 4 or 5) definitions of flourishing; the latter are reported in our Tables as "Flourishing (corrected)."

Table D Results of applying our reproduction of Fredrickson et al.'s mixed effect linear model to the discovery study, compared to Fredrickson et al.'s reported results (their Table 2) for the confirmation study[1].

Well-being model	Well-being dimension	Association *b*	SE	Test statistic	p	VIF
2-dimensional						
	Hedonic well-being	0.536 / *0.085*	0.171 / *0.121*	*t*(58) = 3.13 / *t*(*104*) = *0.70*	.0018 / *.4829*	3.44 / *2.46*
	Eudaimonic well-being	0.135 / *−0.509*	0.175 / *0.125*	*t*(58) = 0.77 / *t*(*104*) = *−4.08*	.4403 / *<.0001*	3.61 / *2.59*
3-dimensional						
	Hedonic well-being	0.573 / *0.098*	0.180 / *0.122*	*t*(57) = 3.17 / *t*(*103*) = *0.80*	.0015 / *.4243*	3.79 / *2.49*
	Psychological well-being	−0.022 / *−0.384*	0.206 / *0.153*	*t*(57) = −0.11 / *t*(*103*) = *−2.50*	.9138 / *.0139*	4.94 / *3.95*
	Social well-being	0.138 / *−0.163*	0.160 / *0.137*	*t*(57) = 0.86 / *t*(*103*) = *−1.19*	.3880 / *.2370*	2.97 / *3.14*
1-dimensional						
	Total well-being	0.612 / *−0.439*	0.106 / *0.086*	*t*(59) = 5.78 / *t*(*105*) = *−5.11*	<.0001 / *<.0001*	1.30 / *1.17*
Categorical						
	Flourishing	0.854 / *−0.615*	0.200 / *0.176*	*t*(59) = 4.27 / *t*(*105*) = *−3.49*	<.0001 / *.0007*	1.21 / *1.17*
	Flourishing (corrected)[2]	1.282 / *−0.693*	0.204 / *0.178*	*t*(59) = 6.27 / *t*(*105*) = *−3.89*	<.0001 / *.0001*	1.36 / *1.17*

[1] The table entries in roman type show the results of applying our reproduction model to the data from the discovery sample (n = 76); those in italics are taken from Fredrickson et al.'s [5] Table 2, which reported the results of applying their model to the confirmation study data (n = 122; hence the different degrees of freedom for the *t*-statistics).

[2] For the corrected "Flourishing" values, the entries in italics correspond to the values that we calculated in our Table C. See also note 2 on that table.

Table E Results of applying our reproduction of Fredrickson et al.'s mixed effect linear model to the discovery study, with participant SOBC1-1293 included or excluded[1].

Well-being model	Well-being dimension	Association *b*	SE	Test statistic	p	VIF
2-dimensional						
	Hedonic well-being	0.536 / *−0.120*	0.171 / *0.078*	*t*(58) = 3.13 / *t*(*57*) = *−1.53*	.0018 / *.4829*	3.44 / *3.64*
	Eudaimonic well-being	0.135 / *0.065*	0.175 / *0.081*	*t*(58) = 0.77 / *t*(*57*) = *0.81*	.1255 / *.4206*	3.61 / *3.87*
3-dimensional						
	Hedonic well-being	0.573 / *−0.157*	0.180 / *0.080*	*t*(57) = 3.17 / *t*(56) = *−1.95*	.0015 / *.0513*	3.79 / *3.91*
	Psychological well-being	−0.022 / *0.173*	0.206 / *0.090*	*t*(57) = −0.11 / *t*(*56*) = *1.92*	.9138 / *.0545*	4.94 / *4.91*
	Social well-being	0.138 / *−0.090*	0.160 / *0.072*	*t*(57) = 0.86 / *t*(*56*) = *−1.24*	.3880 / *.2138*	2.97 / *3.16*
1-dimensional						
	Total well-being	0.612 / *−0.047*	0.106 / *0.047*	*t*(59) = 5.78 / *t*(*58*) = *−1.00*	<.0001 / *.3158*	1.30 / *1.30*
Categorical						
	Flourishing	0.854 / *−0.013*	0.200 / *0.091*	*t*(59) = 4.27 / *t*(*58*) = *−0.15*	<.0001 / *.8830*	1.21 / *1.20*
	Flourishing (corrected)[2]	1.282 / *0.094*	0.204 / *0096*	*t*(59) = 6.27 / *t*(*58*) = *0.97*	<.0001 / *.3304*	1.36 / *1.35*

Note: These analyses assume that the correction of the value of "White" from "4" to "0" for participant SOBC1-1299, discussed in the main text of the present article, has been made.

[1] The table entries in roman type show the results of applying our reproduction model to the data from the discovery sample with participant SOBC1-1293 included (n = 76); those in italics show the results of applying the same model to the same data with participant SOBC1-1293 excluded (n = 75), hence the different degrees of freedom for the *t*-statistics.

[2] See note 2 on our Table C.

References

1. Fredrickson BL, Grewen KM, Coffey KA, Algoe SB, Firestine AM, Arevalo JM, et al. (2013) A functional genomic perspective on human well-being. Proc Natl Acad Sci U S A 110: 13684–13689. doi: 10.1073/pnas.1305419110 PMID: 23898182

2. Keyes CL (2014) Brief description of the Mental Health Continuum Short Form (MHC-SF). Available: http://www.aacu.org/sites/default/files/MHC-SFEnglish.pdf

3. Brown NJL, MacDonald DA, Samanta MP, Friedman HL, Coyne JC (2014) A critical reanalysis of the relationship between genomics and well-being. Proc Natl Acad Sci U S A doi: 10.1073/pnas.1407057111 PMID: 25157145

4. Brown NJL, MacDonald DA, Samanta MP, Friedman HL, Coyne JC (2014) Supporting information for "A critical reanalysis of the relationship between genomics and well-being". Available: http://www.pnas.org/content/suppl/2014/08/21/1407057111.DCSupplemental

5 Fredrickson BL, Grewen KM, Algoe SB, Firestine AM, Arevalo JM, Ma J, et al. (2015) Psychological well-being and the human Conserved Transcriptional Response to Adversity. PLoS ONE 10(3): e0121839. doi: 10.1371/journal.pone.0121839 PMID: 25811656
